# Opposing Roles of BMP and TGF-β Signaling Pathways in Pancreatitis: Mechanisms and Therapeutic Implication

**Published:** 2019-09-04

**Authors:** Yanna Cao, Madeline Drake, Joy Davis, Baibing Yang, Tien C Ko

**Affiliations:** Department of Surgery, The University of Texas Health Science Center, USA

**Keywords:** Bone morphogenetic protein, Transforming growth factor-beta, Gremlin 1, Pancreatitis, Acute, Chronic, Inflammation, Fibrosis, Pancreatic stellate cells, Antibody

## Abstract

Bone morphogenetic proteins (BMPs) comprise a major subgroup of the transforming growth factor (TGF)-β superfamily. They play pivotal roles in embryonic development and tissue homeostasis in adults. Deregulation of BMP and TGF-β signaling contributes to developmental anomalies and multiple diseases. In this mini-review, we focus on BMP signaling in inflammatory disorders of the pancreas, acute and chronic pancreatitis, in contrast to TGF-β signaling. We then discuss molecular mechanisms that interact with and connect between the BMP and TGF-β signaling pathways. Lastly, we review potential implications of these molecular mechanisms for therapeutic development. In summary, BMP signaling pathway plays different roles during pancreatitis disease development, and the antagonism between BMP and TGF-β signaling can be manipulated for therapeutic development against pancreatitis.

## Introduction

Bone morphogenetic proteins (BMPs) constitute a major subgroup of the transforming growth factor (TGF)-β superfamily, comprising 18 (several also named as growth differentiation factors) out of the total 33 members [1]. The TGF-β/BMP superfamily plays pivotal roles in embryonic development and tissue homeostasis in adults. Deregulation of the TGF-β/BMP signaling contributes to developmental anomalies and multiple diseases [1,2]. The TGF-β/BMP superfamily signals through a complex of membrane-associated serine/threonine kinase receptors. In the canonical signaling pathway, BMP ligand binds to BMP receptors type I (BMPR1) and type II (BMPR2) on the cell surface and subsequently activates intracellular mediators Smad1/5/8 via phosphorylation [3]. In parallel, TGF-β binds to TGF-β receptors TβRI and TβRII and activates Smad2/3. The phosphorylated Smad1/5/8 or pSmad2/3 form complexes with Smad4, which translocates to the nucleus to regulate transcription of target genes [4,5]. Activation of specific Smads may lead to distinct or opposing biological outcomes, most notably in disease states [3,6,7]. Both BMP and TGF-β also activate other non-canonical signaling pathways [8,9]. BMP signaling can be regulated at the extracellular, membrane, and intracellular level. The most investigated molecules that negatively regulate BMP signaling extracellularly are the BMP antagonists, which sequester BMPs and prevent their interaction with the cell surface receptors. Over 12 members of endogenous BMP antagonists have been identified to date [3,10].

Lines of evidence demonstrate that BMP signaling has pro-inflammatory properties in bronchial epithelial cells during airway inflammation [11], in activated endothelial cells [12], and in atherosclerotic arteries [13]. Paradoxically, BMP signaling has anti-fibrogenic functions in several organs, including kidneys, lungs, and liver [14-16]. However, the role of BMP signaling in the pancreas, specifically within pancreatic inflammatory disease processes, is unclear.

In recent years, our group has focused on the role of BMP signaling in acute (AP) and chronic pancreatitis (CP), the highly debilitating and painful inflammatory diseases of the pancreas. While a majority of AP cases self-resolve, approximately 16% progress to CP after repeated episodes of AP (RAP), mainly in patients with risk factors like alcohol and tobacco use [17,18]. CP is the #1 cause of Type 3c Diabetes [19], and a major risk factor for pancreatic cancer, one of the most lethal cancer types [20]. Unfortunately, the standard care for pancreatitis lacks specific pharmacological therapies, and remains primarily supportive. Thus, there is a pressing need to identify key mechanisms contributing to the disease development and progression, in order to develop innovative therapeutic strategies for blocking and reversing the pancreatic destruction associated with CP; ultimately, reducing the risk for developing diabetes and pancreatic cancer in these patients.

To understand the role of BMPs in AP, we utilized experimental animal and cell models, and revealed that BMP/Smad1/5 signaling is activated; inhibition of BMP signaling attenuates the disease severity, indicating a pro-inflammatory role of BMP signaling in AP [21]. The role of TGF-β signaling is controversial, with both pro-inflammatory and anti-inflammatory roles reported [22]. However, due to a lack of availability of most human AP samples, our study regarding BMP signaling is only proof-of-concept. Much work is needed to establish clinical relevance by identifying secreted factors of the TGF-β/BMP superfamily from patient blood and pancreatic juice, in addition to further study of the mechanisms in experiment models.

Several studies from our group have demonstrated opposing roles of BMP and TGF-β in CP. For instance, in pancreatic stellate cells, the key executive cells in pancreatic fibrosis, BMP2 alone does not induce extracellular matrix (ECM) expression but inhibits TGF-β induced-ECM production [23]. Knockout of BMPR2 in mice exacerbates CP, leading to enhanced inflammation and fibrosis, two hallmarks of CP [24]. These findings reveal a protective and anti-fibrogenic role of BMP signaling in CP, in contrast to the pro-fibrogenic TGF-β signaling [25,26].

**Figure 1:**
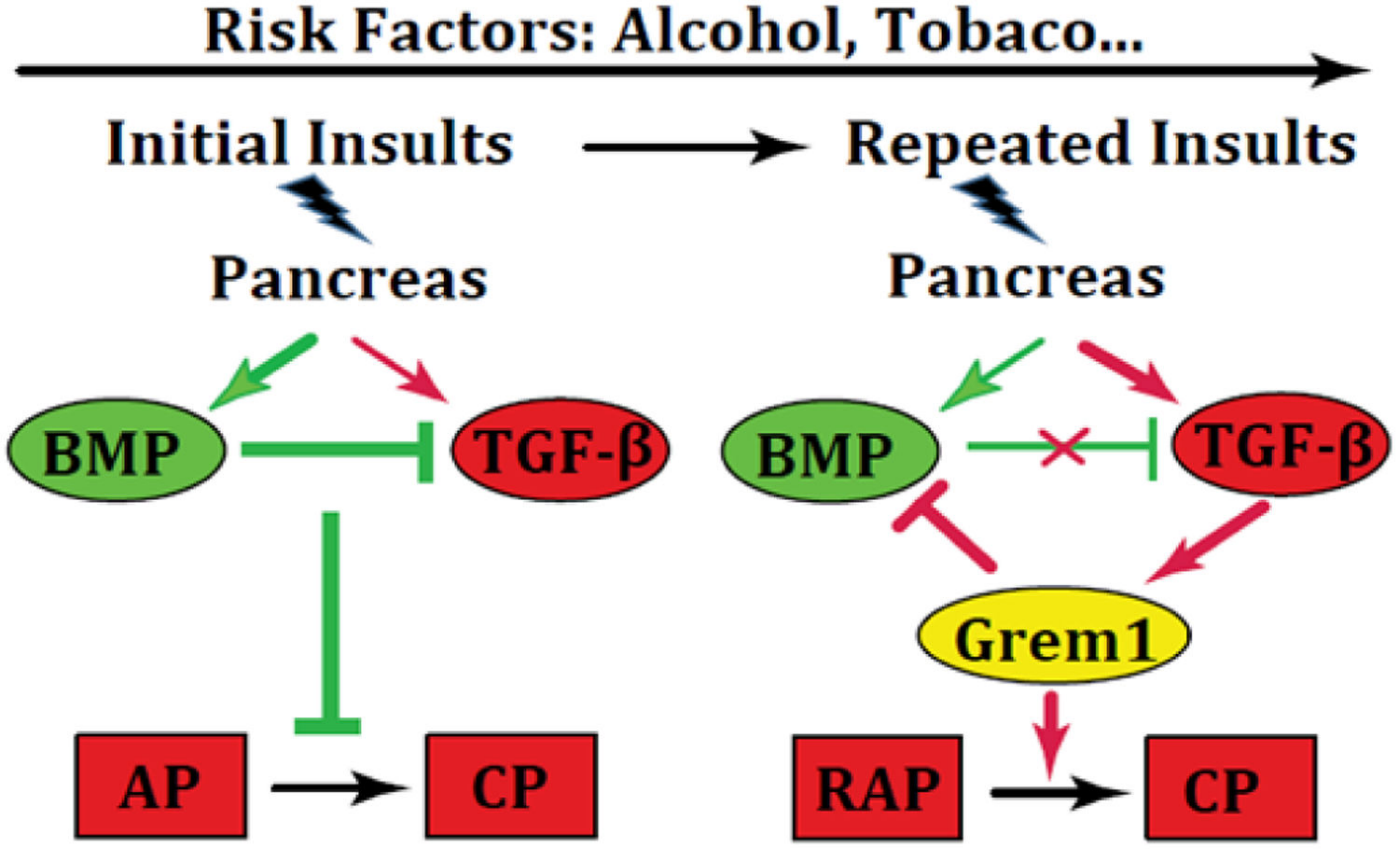
Proposed interaction between BMP, TGF-β, and Grem1 During AP to CP Progression.

To search for molecular links between BMP and TGF-β, we focus on Gremlin1 (Grem1), a BMP antagonist, which has reported pro-fibrogenic function in several organs [15,27,28]. We demonstrated increased levels of Grem1 in human and mouse CP, associated with elevated TGF-β. TGF-β can induce Grem1, and Grem1 can block BMP2 induced Smad1/5 signaling in pancreatic stellate cells. Knockout of Grem1 in mice attenuates pancreatic fibrosis [29]. These findings propose a feed-forward loop between TGF-β, Grem1, and BMP, in which Grem1 may act as a nodal point between the pro-fibrogenic TGF-β and the anti-fibrogenic BMP signaling pathways. Thus, the level of Grem1 expression may define the disease progression from AP, RAP, to CP (Figure 1].

Overall, temporal changes of the TGF-β/BMP signaling molecules and Grem1 during disease progression from AP to CP [23,24,29,30], provide rationale for further translational study. Since TGF-β has a broad spectrum of biological functions, systemic TGF-β blockade may yield unexpected or detrimental effects. To circumvent these effects, modulation of TGF-β downstream mediators and of the opposing BMP signaling that desensitize cellular responses to TGF-β or antagonize TGF-β signal transduction should be explored. Thus, the therapeutic goal is to restore or up-regulate the anti-fibrogenic BMP signaling pathway and to counteract the pro-fibrogenic TGF-β signaling pathway. This can be executed by time-dependently administering the Grem1 neutralization antibody [31] or specific microRNAs [32,33] that can inhibit Grem1 as well as release suppression on BMP signaling, or in combination with the specific small molecules or peptides that can activate BMP signaling [34,35].

## Conclusion

Studies on the different roles and the reciprocal regulation of BMP and TGF-β not only advance our knowledge on how the same superfamily members regulate each other’s functions in diseased states, but also provide insights on how the antagonistic roles of BMP and TGF-β signaling can be modulated as therapeutic approaches.

## Abbreviations:

BMP: Bone Morphogenetic Protein
TGF-β: Transforming Growth Factor-Beta
microRNA: Micro Ribonucleic Acid
ECM: Extracellular Matrix
AP: Acute Pancreatitis
CP: Chronic Pancreatitis
